# Walking speed at the acute and subacute stroke stage: A descriptive meta-analysis

**DOI:** 10.3389/fneur.2022.989622

**Published:** 2022-09-26

**Authors:** Sophie Tasseel-Ponche, Arnaud Delafontaine, Olivier Godefroy, Alain P. Yelnik, Pierre-Louis Doutrellot, Charline Duchossoy, Marie Hyra, Thibaud Sader, Momar Diouf

**Affiliations:** ^1^Department of Physical Medicine and Rehabilitation, Amiens University Hospital, Amiens, France; ^2^Laboratory of Functional Neurosciences (EA 4559), Amiens University Hospital, Amiens, France; ^3^CIAMS, Paris-Saclay University, Orsay, France; ^4^CIAMS, Orléans University, Orléans, France; ^5^Department of Neurology, Amiens University Hospital, Amiens, France; ^6^Physical Medicine and Rehabilitation Department, Hôpital Lariboisière-F. Widal AP-HP, Paris, France; ^7^INSERM U1153 - CRESS EpiAgeing, Paris University, Hôtel-Dieu, Paris, France; ^8^Department of Biostatistics, Amiens University Hospital, Amiens, France

**Keywords:** stroke, gait disorders, walking speed, short-distance, long-distance, meta-analysis

## Abstract

Gait disorders are one of the leading patient complaints at the sub-acute stroke stage (SSS) and a main determinant of disability. Walking speed (WS) is a major vital and functional index, and the Ten-Meter Walk Test is considered the gold standard after stroke. Based on a systematic review of the literature, studies published between January 2000 and November 2021 were selected when WS was reported (ten-meter walk test for short distance and/or 6-min walking distance for long distance) within 6 months following a first ischemic and/or hemorrhagic stroke (SSS) in adults prior to receiving specific walking rehabilitation. Following PRISMA guidelines, a meta-analysis was conducted on two kinds of WS: the principal criterion focused on short-distance WS (ten-meter walking test) and the secondary criteria focused on long-distance WS (6-min test) and meta-regressions to study the association of WS with balance, cognitive disorders and autonomy. Nine studies comprising a total of 939 data on post-stroke patients were selected. The weighted average age was 61 years [95% IC [55-67] and males represented 62% ± 2.7 of patients [57-67]. Average short-distance WS was 0.36 ± 0.06 m.s^−1^ [95% CI (0.23–0.49)]. Average long-distance WS was 0.46 ± 0.1 m.s^−1^ [95% CI (0.26–0.66)]. The funnel plot revealed asymmetry of publication bias and high heterogeneity of the nine studies (*I*^2^ index 98.7% and *Q*-test *p* < 0.0001). Meta-regressions of secondary endpoints could not be performed due to a lack of study data. At the SSS, WS would be lower than data in general population published in literature, but above all, lower than the WS required for safe daily autonomy and community ambulation after stroke. WS must be a priority objective of stroke rehabilitation to increase walking function but also for survival, autonomy, social participation and health-related quality of life.

## Introduction

Gait disorders are one of the leading sequelae (1, 2) and a major determinant of autonomy and disability (3–5) in stroke survivors. Reported prevalence of gait disorders ranges between 54 and 80% (2, 6, 7). After stroke, gait ability requires greater postural, motor and cognitive control (8–10), and especially executive functions that are impaired in more than 50% of poststroke patients (11–13). Gait disorders are associated with a high risk of falls (with 70% of patients experiencing a fall during the first year after stroke), impact autonomy and have socioeconomic consequences (14–16).

Walking speed (WS) is an important functional measure for vital status and gait ability after stroke (17–21). The comfortable ten-meter walking test is the gold standard assessment of short-distance WS in the stroke population (22). WS >0.80 m.s^−1^ (meters per second) is usually considered to be necessary for safe ambulation in daily activities, such as safely crossing a street (23, 24). WS >0.83 m.s^−1^ is necessary to optimize the energy cost during walking (10). Improvement in WS has been reported between three and 18 months after stroke, while other sensorimotor impairments assessed by the Fugl-Meyer Assessment (FMA) and the Barthel Index (BI) preferentially improve between 6 weeks and 3 months (25). Comfortable gait speed measured using the ten-meter walking test consistently relates to balance and strength impairments, mobility limitations across settings, daily living activities, physical activity and other meaningful activities relevant to ambulatory patients at the chronic stage after stroke (26). Although these data support the functional importance of WS from the sub-acute stage, no systematic review has established the WS of stroke patients, especially due to the improvement of the vital and functional prognosis thanks to thrombectomy and thrombolysis worldwide (27, 28). In the literature, a previous meta-analysis evaluated WS at the chronic poststroke phase but not in the early phase [acute and sub-acute stroke stage (ASSS)] (25).

The main objective of this study was to assess short-distance WS according to a systematic review of the literature and a meta-analysis of survivor data at the ASSS. We chose the WS on short walking distance for main objective of the metanalysis because at the acute and subacute stage of strokes, patients are beginning to walk indoors for a short distance. The secondary objectives were to study long-distance WS and the relationships between WS and balance disorders, cognitive status and degree of dependence within 6 months after stroke. We chose the WS on long distance for secondary outcome of the metanalysis to explore a complementary aspect of the performance of WS.

## Materials and methods

### Eligibility criteria

Eligibility criteria were WS data from studies published between 2000 and 2021 to obtain a recent homogeneous population from stroke units after introduction of the thrombolysis and thrombectomy because these treatments have revolutionized poststroke prognosis (27, 28), regardless of sample size to limit selection bias, with assessment of WS (ten-meter walking test for short distance and/or 6-min walking distance for long distance) within 6 months following a first ischemic and/or hemorrhagic stroke, and prior to any specific walking rehabilitation. We chose to focus on the ten-meter walking test because it is the only test with evidence of excellent reliability and construct validity in people with acute and subacute stroke (ASS) (22).

We excluded studies reported as abstract-only without full-text available, non-English articles, studies conducted on animal or on pediatric samples, studies that included patients unable to walk (considered as WS of 0 m.s^−1^) or patients selected according a defined WS (i.e., maximal WS) or walking ability categories [i.e., FAC: Functional Ambulation Classification (FAC)], studies assessing barefoot WS or patients with weight or height limitations to use a walking device (i.e., exoskeleton or treadmill) and published reports without original data. When data from the same cohort were reported in several papers, we selected the study reporting the largest sample meeting our eligibility criteria.

### Information sources procedure

This meta-analysis was conducted according to PRISMA guidelines (29). Studies were obtained from scientific databases, including PubMed and Cochrane Library. The literature search was conducted using selected English language publications databases to explore published biomedical data on strokes.

### Search strategy

The literature search was performed using the following terms and keywords: (speed OR distance) AND (gait OR walk^*^) AND (stroke OR (cerebral AND infarct) OR (cerebral AND hemorrhag^*^) OR (cerebral AND ischem^*^)) AND (acute OR recent) AND (human AND adult) AND (“2000/01/01” [PDAT]: “2021/11/31” [PDAT]) AND (cohort studies OR group). The search engine Pubmed was chosen to provide free access to Medline^®^ contains journal citations and abstracts for biomedical literature from around the world and Cochrane to search meta-analysis on walking speed after stroke. To ensure literature saturation, reference lists of included studies and relevant reviews identified through the search were analyzed by the authors.

### Selection and data collection screening

Two reviewers (AD, STP) conducted the search, selected studies and included reporting on WS investigated in patients during the first 6 months following ischemic or hemorrhagic stroke. In event of a disagreement between the reviewers regarding study inclusion or exclusion, a third reviewer's opinion (TS) was sought before including data in the meta-analysis. When the majority of the reviewers agreed, the study was included in meta-analysis according to PRISMA guidelines (29). The data collection process protocol was prepared according to a data collection table.

### Data items and extraction

The data collection table included the scale used and the following data from selected articles: mean age and sex ratio, time since stroke and type of stroke, department (neurology: stroke unit or rehabilitation) of inpatients or community-dwelling patients (ambulatory care), exclusion or inclusion of patients with comorbidities, quantitative gait assessments (ten-meter walking test or 6-min walking test), the use of a qualitative video system or quantified gait analysis system, functional walking assessment, balance assessment, cognitive assessment and autonomy status. The time chosen for the assessments included was the first 6 months after stroke, before any rehabilitation research protocol but standard physical therapy was not an exclusion criterion.

### Study risk of bias assessment

To decrease risk of study bias, three reviewers (AD, STP, TS) assessed each study to obtain a majority in case of disagreement. In the event of questions, one of the two reviewers (AD or STP) asked the two other reviewers (AD, STP, TS) to analyze the problematic study independently.

The problematic article was included or excluded based on the opinion of the majority of reviewers according to PRISMA guidelines (29). No automation tool was used in the process. We attempted to acquire any missing information to limit bias. For studies comparing different types of care or interventions, only the initial data prior to the intervention were included in this analysis. The review was not registered.

### Synthesis methods and statistical analysis

The main criterion was the ten-meter walking test (short-distance WS) according to a meta-analysis of survivor data at the ASSS. The data collection table collected data from the selected articles. Average and/or median ten-meter WS expressed or calculated in meters per second (m.s^−1^) and their variances were first identified for each study. When variances were not reported, standard error was estimated as one-sixth of the study's WS range. The WS of each study were represented in a forest plot. The pooled values were calculated using the random-effect model of DerSimonian and Laird (30) and an ~95% confidence interval based on within-study variance and between-study variance of WS.

The secondary criteria were (i) meta-regressions of WS according to balance disorders [Berg Balance Scale (BBS)], cognitive status [Mini Mental State Examination (MMSE)] and autonomy [Barthel Index (BI)] within 6 months after stroke; and (ii) the 6-min walking test (long-distance WS) according to a meta-analysis of survivor data at the ASSS with a limit of statistical significance of *p* < 0.05.

The heterogeneity of WS between studies was evaluated using the *I*^2^ index and the *Q* test (31). Heterogeneity was interpreted as usual: *I*^2^ < 25%, no heterogeneity; 25% ≤ *I*^2^ ≤ 50%, moderate heterogeneity; and *I*^2^ > 50%, high heterogeneity. A *p*-value <0.05 of the *Q* test was interpreted as statistically significant heterogeneity. The presence of a potential publication bias or heterogeneity was examined using Egger's method and a funnel plot (32). Statistical analysis was performed using RStudio© software version 1.0.143.

## Results

### Study selection

**Figure 1:**
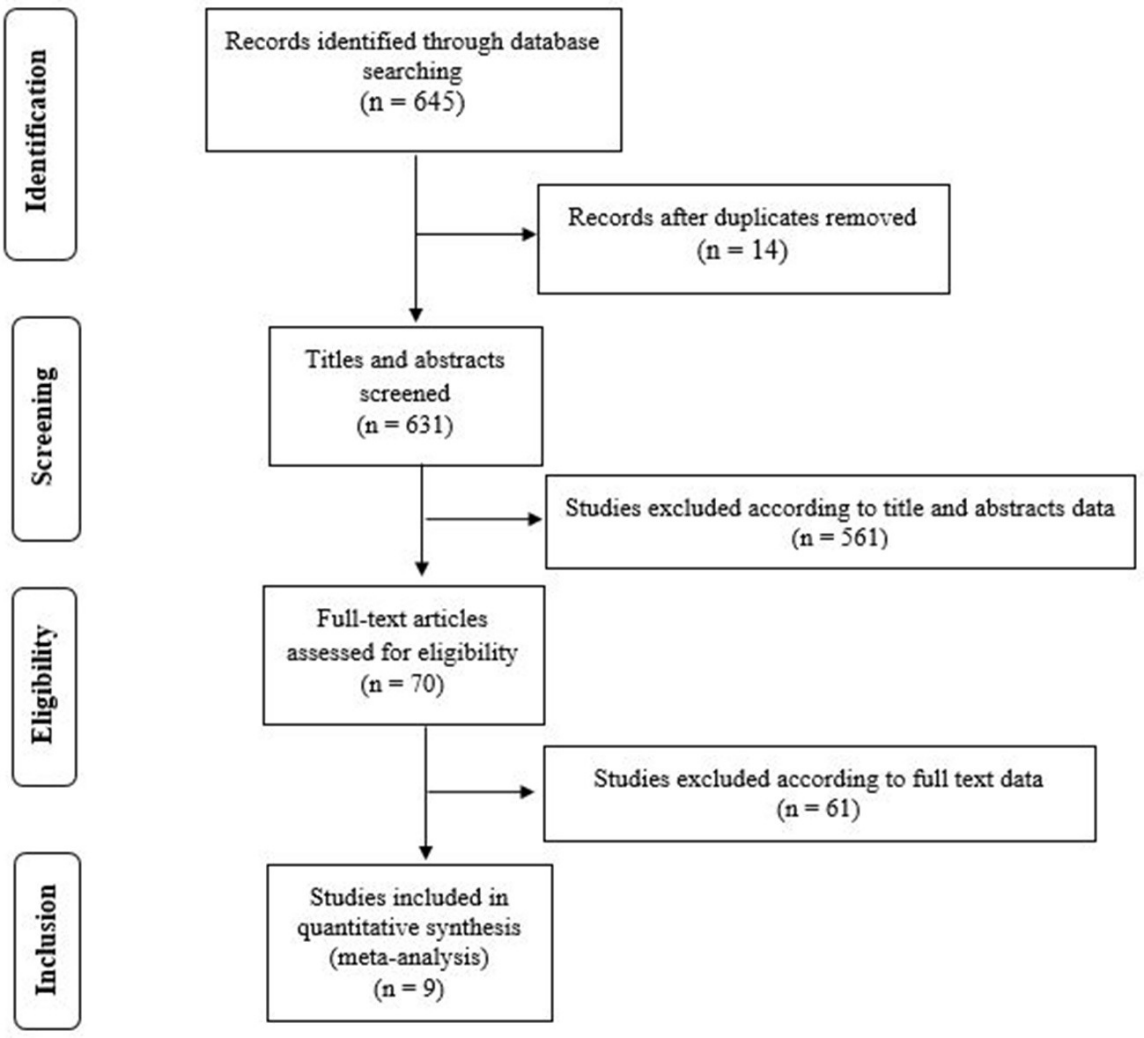
PRISMA flow diagram summarizing the article selection process.

The literature search revealed a total of 645 articles (Figure 1). Following abstract analysis, 70 full articles were analyzed and nine studies meeting inclusion criteria were selected for meta-analysis as summarized in Table 1.

**Table 1:** Clinical characteristics of the nine studies included in the meta-analysis of short-distance WS.

**Studies**	***N***	**Age (years)**	**Time post S (days)**	**I/H/U stroke (%)**	**Type of care N/R/A**	**WS (m.s^−1^)**	**FAC**	**BBS**	**MMSE**	**BI**
Chua et al. (33)	106	61.4 (10.5)	28.5 (12.7)	MD	R	0.24 (0.15)	1 [0–3]	MD	24.4 (4.1)	48.7(35.2)
Oostra et al. (34)	44	52 (12.4)	124.5 (76.5)	63.6/36.4/0	R	0.32 [0.34]	MD	MD	MD	MD
Conesa et al. (35)	69	48 (11)	72 (36)	47.8/52.2/0	R	0.17 (0.22)	1.3 (1.23)	MD	MD	MD
Brincks and Nielson (37)	13	61 [38–71]	27 [9–79]	100/0/0	R	0.24 [0.03–0.5]	MD	MD	MD	MD
Tilson et al. (15)	408	62 (12.7)	63.8 (8.5)	71/17.2/11.8	A	0.38 (0.22)	MD	36 (14)	26.1 (3.5)	MD
Mak and Lau (38)	30	70.8 (10.15)	12.8 (5.5)	100/0/0	R	0.29 (0.19)	MD	40 (13.2)	MD	MD
Persson et al. (39)	96	76 [47–94]	7 (–)	89.6/10.4/0	N	0.71 [0.1–1.6]	MD	42 [0–56]	MD	MD
Kollen et al. (36)	81	64.3 (10.8)	8.2 (2.7)	100/0/0	R	0.04 (0.1)	0.89 (1)	MD	26.6 (2.3)	6.90 (3.7)
Duncan et al. (40)	92	69.3 (10.2)	75.5 (27.9)	90.2/9.8/0	A	0.65 (0.3)	MD	MD	MD	MD
Number of valid studies	**9**	**9**	**9**	**8**	**9**	**9**	**3**	**3**	**3**	**2**
Number of subjects	**939**	**939**	**939**	**833**	**939**	**939**	**256**	**534**	**595**	**187**
	**100%**	**100%**	**100%**	**86%**	**100%**	**100%**	**26.3%**	**55%**	**61.3%**	**19.3%**

Data expressed as mean (standard deviation) or interquartile range [IQR] or minimum - Maximum [min-Max]. MD, missing data; N, number of subjects; S, stroke; I, ischemic stroke; H, hemorrhagic stroke; U, undetermined stroke; N, neurology unit; R, rehabilitation unit; A, ambulatory care; WS, walking speed; FAC, Functional Ambulation Classification; BBS, Berg Balance Scale; MMSE, Mini Mental State Examination score; BI, Barthel Index; N, acute stroke unit; R, rehabilitation department; A, ambulatory care. Pooled values are presented as mean for continuous variables (age, time post stroke, BBS, MMSE, BI) and as percentage for categorical variables (ischemic, hemorrhagic or undetermined strokes).

### Studies, population characteristics and data

The nine studies included in the meta-analysis of short-distance WS represented a total of 939 patients (Tables 1, 2). The weighted average age was 61 years (95% IC [55-67]), and majority of patients were male (62% ± 2.7 [57-67]). The average assessment time after stroke was 48.1 days. Ischemic stroke was predominant, accounting for 82.8% of the population. Most studies excluded patients with persistent comprehension disorders and a contraindication to participation in walking rehabilitation programs such as hemodynamic instability, previous stroke, another neurological disease and comorbidities such as disabling rheumatological disease. Six studies assessed gait performance conducted in rehabilitation departments (33–38), two in ambulatory care (15, 40) and one in stroke units (39). Three studies used a qualitative video system or a quantified gait analysis system (35, 37, 38) (Table 1).

**Table 2:** Parameters of ten-meter walk test for short distance WS.

**Studies**	***N***	**WS (m.s^−1^)**	**Distance (acceleration—recording—deceleration) m**	**Video recording (Yes—No)**	**Number of training—trials**	**Mean (M) or best (B) trial or single test (1)**	**Reference of assessment**
Chua et al. (33)	106	0.24 (0.15)	/	/	/−2	B	(41)
Oostra et al. (34)	44	0.32 [0.34]	/	/	/−1	1	(42)
Conesa et al. (35)	69	0.17 (0.22)	/	Yes	/−3	M	(43)
Brincks and Nielson (37)	13	0.24 [0.03–0.5]	2−6−2	Yes	/—/	/	/
Tilson et al. (15)	408	0.38 (0.22)	3−10−3	/	/—/	/	(44)
Mak and Lau (38)	30	0.29 (0.19)	/	Yes	1−3	B	(45)
Persson et al. (39)	96	0.71 [0.1–1.6]	/	/	/	/	(45)
Kollen et al. (36)	81	0.04 (0.1)	/	/	0−3	/	/
Duncan et al. (40)	92	0.65 (0.3)	2−8−2	/	1−3	M	(43)

Data expressed as mean (standard deviation) or interquartile range [IQR] or minimum - Maximum [min-Max]. /, missing data; *N*, number of subjects; WS, walking speed; m, meter; V, video recording; B, best trial; M, mean trials.

### Risk of bias in studies

**Figure 2:**
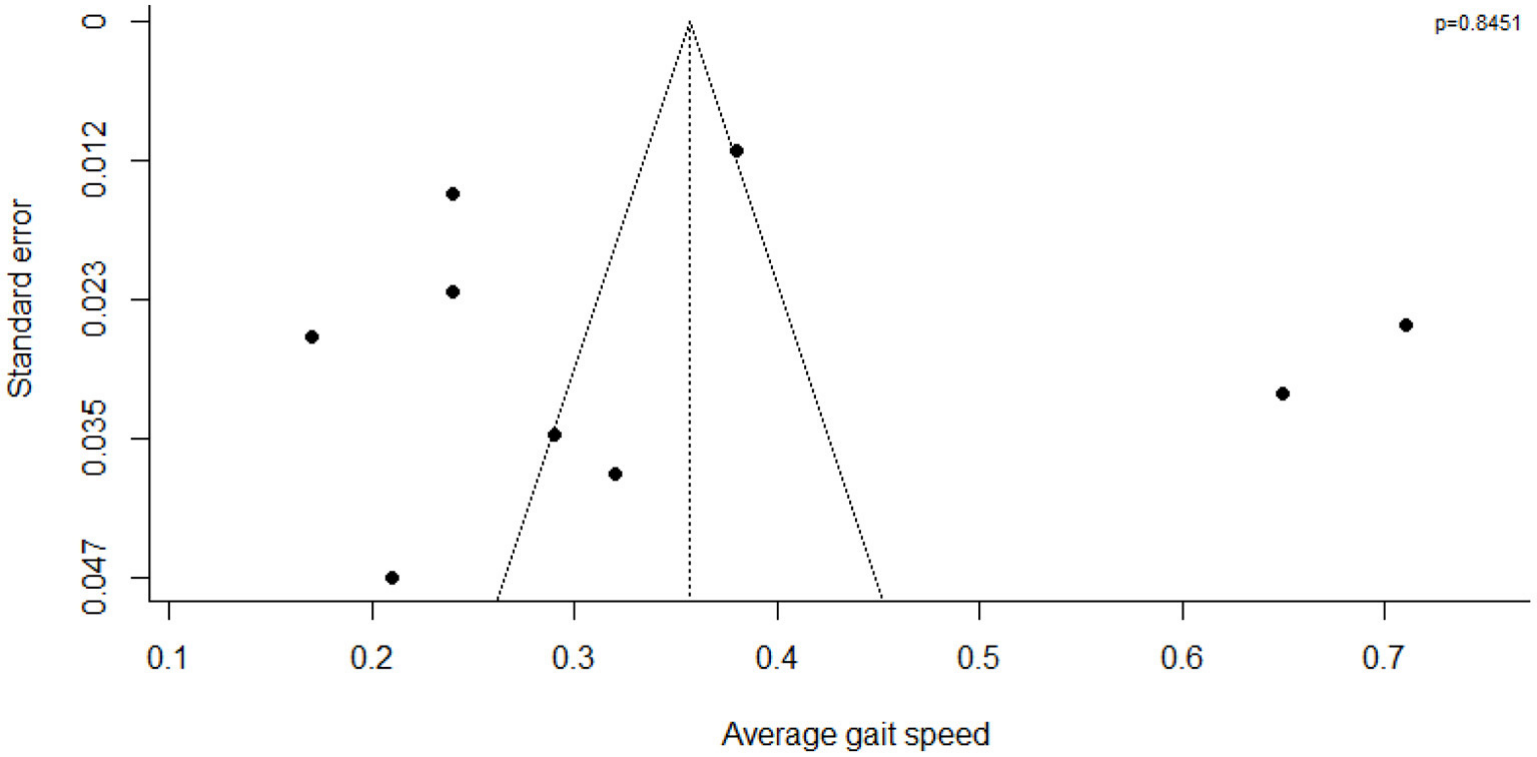
Funnel plot of walking speed (m.s^−1^) in the nine studies of the meta-analysis.

The funnel plot (Figure 2) revealed asymmetry of publication bias and the large proportion of studies outside the 95% interval suggested high heterogeneity of the nine studies.

**Figure 3:**
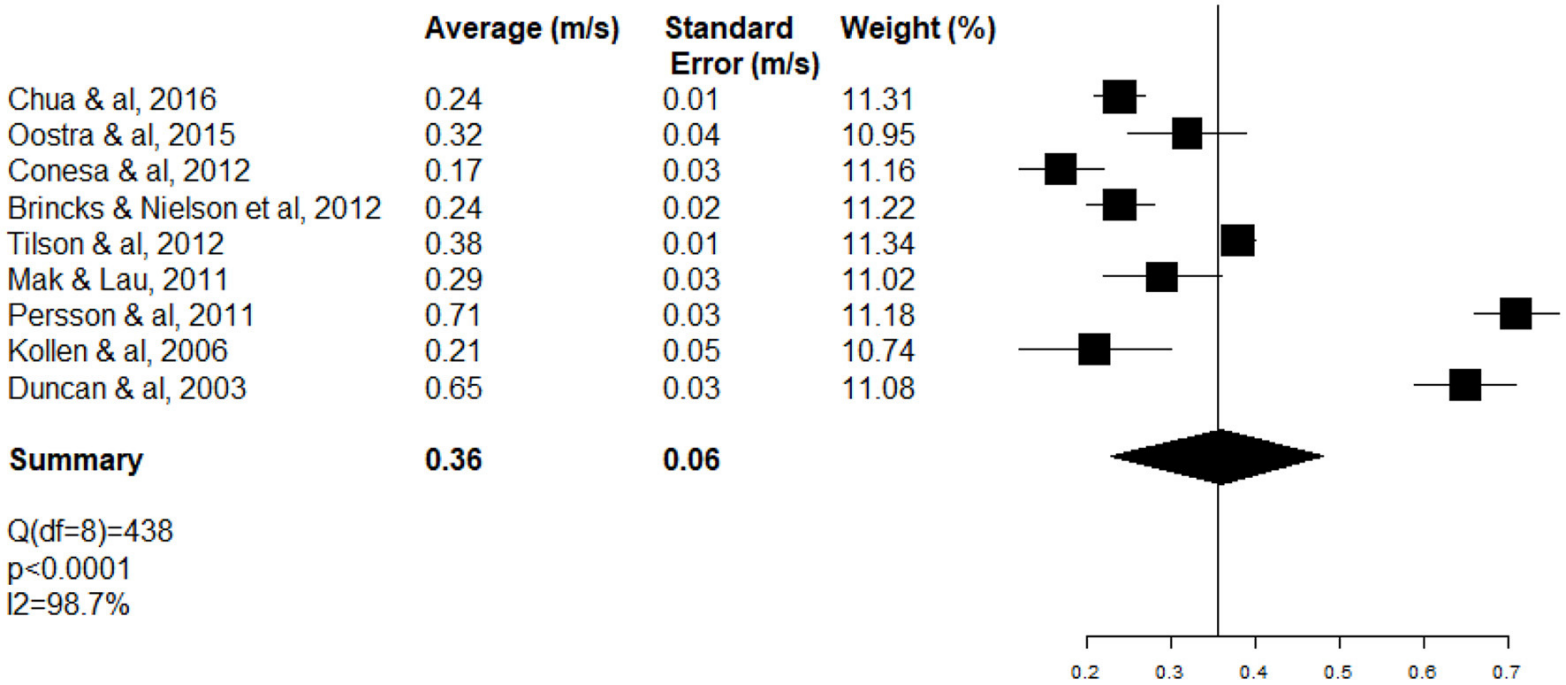
Forest plot of short-distance WS at the acute and subacute stages of stroke (ASSS).

Variation of WS resulting in marked large heterogeneity (*I*^2^ 98.7%; test *Q* significant *p* < 0.0001) (Figure 3).

### Primary objective: Results of meta-analysis of short-distance WS

After meta-analysis of the nine studies, short-distance WS was 0.36 ± 0.06 m.s^−1^ [95% CI (0.23–0.49)] at the ASSS (Figure 3).

### Secondary objectives

Meta-regressions of secondary endpoints could not be performed due to a lack of study data on secondary criteria: three studies assessed cognitive functions according to the Mini Mental State Examination (MMSE) (15, 33, 36), three studies assessed functional walking abilities according to functional ambulation categories (FAC) (33, 35, 36) and balance according to the Berg Balance Scale (BBS) (15, 38, 39), and two studies assessed autonomy according to the Barthel Index (BI) (33, 36).

**Figure 4:**
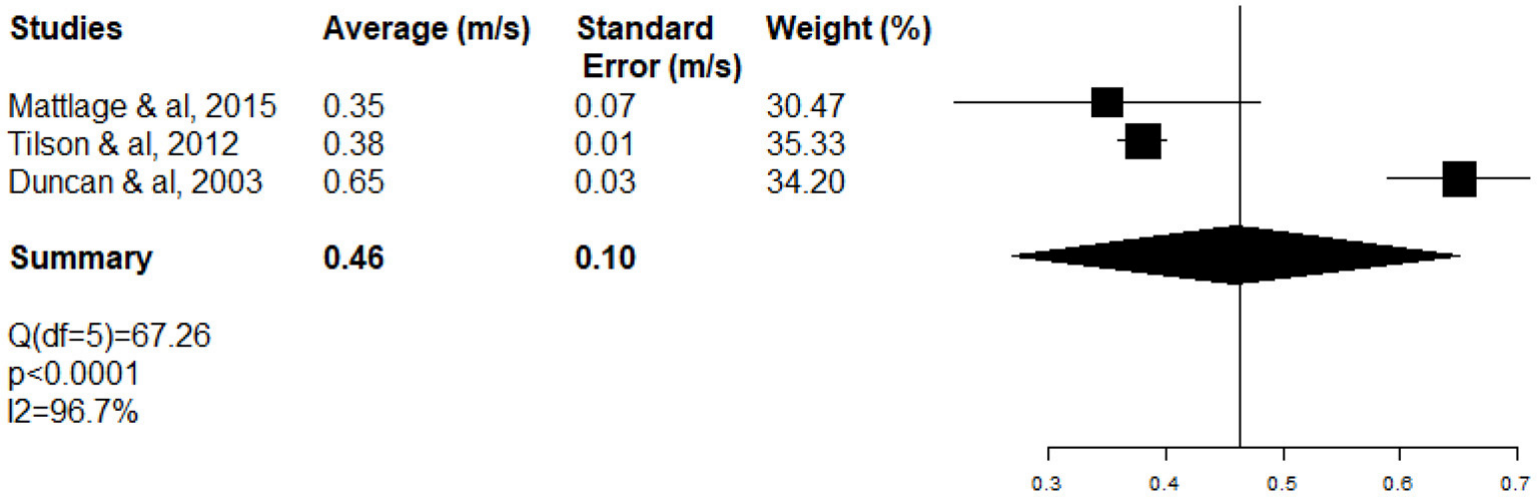
Forest plot of long-distance WS at the acute and subacute stages of stroke (ASSS).

The meta-analysis of long-distance WS included three studies (15, 40, 46) (see Table 3), assessing long-distance WS (0.46 ± 0.10 m.s^−1^ [95% CI (0.26–0.66)]) with the 6-min walking test at the ASSS (Figure 4).

**Table 3:** Clinical characteristics of the three studies included in the meta-analysis of long-distance WS.

**Studies**	**Nb**	**Age (years)**	**Time post S (days)**	**I/H/U stroke (%)**	**Type of care N/R/A**	**WS (m.s^−1^)**	**FAC**	**BBS**	**MMSE**	**BI**	**WS assessment^a^**
Mattlage at al. (46)	32	56.5 (12.7)	2	MD	N	0.35 (0.37)	MD	MD	MD	MD	100/R/No (47)
Tilson et al. (15)	408	62 (12.7)	63.8 (8.5)	71/17.2/11.8	A	0.35 (0.22)	MD	36 (14)	26.1 (3.5)	MD	100/R/No (48)
Duncan et al. (40)	92	69.3 (10.2)	75.5 (27.9)	90.2/9.8/0	A	0.65 (0.3)	MD	MD	MD	MD	MD/R/MD (49)
Number of valid studies	**3**	**3**	**3**	**2**	**3**	**3**	**0**	**1**	**1**	**0**	**3**
Number of subjects	**939**	**939**	**939**	**833**	**939**	**939**	**256**	**534**	**595**	**187**	**939**
	**100%**	**100%**	**100%**	**86%**	**100%**	**100%**	**26.3%**	**55%**	**61.3%**	**19.3%**	**100%**

a Walking corridor length (feet)/rest allowed: R or no/Training or practice trials: T or No.

Data expressed as mean (standard deviation) or interquartile range [IQR] or minimum - Maximum [min-Max]. MD, missing 179 data; Nb, number of subjects; S, stroke; I, ischemic stroke; H, hemorrhagic stroke; U, undetermined stroke; N, neurology unit; Re, rehabilitation unit; A, ambulatory care; WS, walking speed; FAC, Functional Ambulation Classification; BBS, Berg Balance Scale; MMSE, Mini Mental State Examination score; BI, Barthel Index; R, rest; T, training or practice trials.

Pooled values are presented as mean for continuous variables (age, time post stroke, BBS, MMSE, BI) and as percentage for categorical variables (ischemic, hemorrhagic or undetermined strokes).

**Figure 5:**
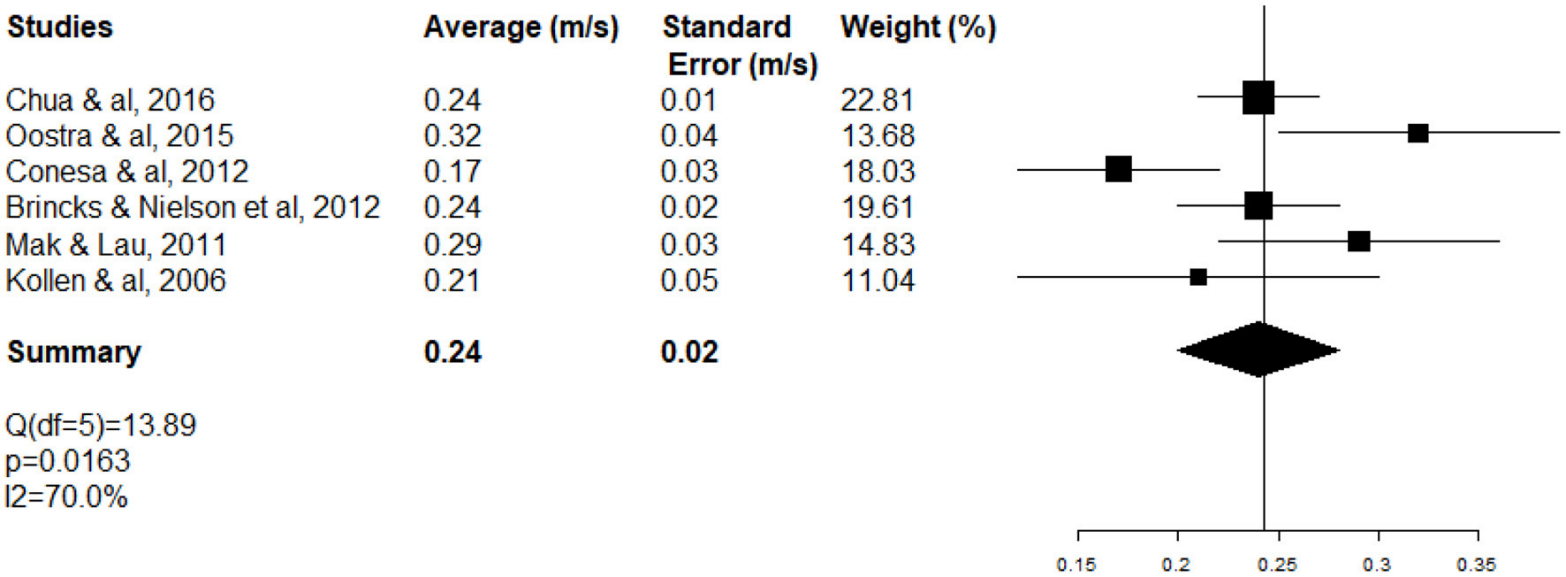
Forest plot of WS of rehabilitation inpatients at the acute and subacute stage of stroke (ASSS).

The high heterogeneity of WS meta-analysis (*I*^2^ 98.7%; test *Q* significant *p* < 0.0001) (Figure 3) can be explained partially by the fact that inpatients (neurology and rehabilitation departments) and outpatients (ambulatory care) were included at different stages (ASSS). One-third of heterogeneity of WS (Δ *I*^2^ 29%) decreased with the subgroup of rehabilitation patients (*I*^2^ 70%; test *Q* significant *p* = 0.0163) (Figure 5).

## Discussion

To our knowledge, this is the first meta-analysis of WS at the ASSS (within 6 months). The short-distance WS of 0.36 m.s^−1^ is consistent with recent literature reporting very short-distance WS of 0.4 m.s^−1^ on the five-meter walking test (50, 51), higher than 0.25 m.s^−1^ reported at the SSS and higher than 0.23 m.s^−1^ reported in a subgroup of hemiparetic patients at the chronic poststroke phase (25). This discrepancy is likely due to the selection of more severely impaired patients in contrast to this meta-analysis, which was performed on post-stroke patients able to walk ten meters without human aid. Moreover, patients at the subacute stage of stroke beneficed of physical therapy except any rehabilitation research protocol, which could overestimate WS in this meta-analysis.

A recent systematic review (52) identified predictors of regaining independent walking at 3 months after stroke as younger age, intact corticospinal tract, good leg strength, absence of cognitive impairment and neglect, continence, good sitting and independence in daily life activity (52). However, only three factors (i.e., younger age, continence and good sitting) persisted at 6 months after stroke and their links to WS were not studied (52).

By comparison, according to a metanalysis of (53), the average WS for general population of the same age, 61 years, is expected to be 1.24 m.s^−1^ in women and 1.34 m.s^−1^ in men (53). This would indicate an estimation about 71–73% decrease of short-distance WS at the ASSS, in women and in men, respectively. An average short-distance WS of 0.36 m.s^−1^ corresponded to one-third of the required WS to safely cross streets (1.2 m.s^−1^) and one-half of the required WS for community ambulation (0.8 m.s^−1^) (2, 23). Our results indicate that ASS patients experience major gait slowing, which is responsible for impaired quality of life (24, 54). Long-distance WS was not significantly greater than short-distance WS (respectively 0.46 m.s^−1^ vs. 0.36 m.s^−1^, *p* > 0.05) and higher than the literature (0.39 m.s^−1^). Although short- and long-distance WS seem to explore two complementary aspects of walking: indoor walking and outdoor walking, they are highly correlated (6-min walking test and ten-meter walking test: *r* = 0.89) (55). Post-stroke, 6-min walking test was more correlated with speed walking (*r* = 0.89) than cardiorespiratory function (VO2 max) (*r* = 0.66) (55, 56). Stroke engine proposes 6-min walking test to explore community mobility (50).

This review studied WS in all kinds of stroke survivors at the ASSS: both hospitalized inpatients (stroke unit and rehabilitation unit) and ambulatory patients. The percentage of rehabilitation inpatients was significant, so the present study population was younger (61 years) in term of representativeness than the mean age of stroke patients (73.5 years) and the sex ratio included fewer women than in the literature (57). The proportion of ischemic stroke was similar to the literature (57). These characteristics are usual in stroke patients selected for such studies, especially when gait skills are required to assess WS because a poor functional prognosis is linked to the age of stroke patients (58, 59).

The main limits of this study were a marked heterogeneity of the meta-analysis, which is likely due to inclusion of patients with different stroke severities, at different stroke stages, and assessed in different settings (stroke units, rehabilitation departments or ambulatory care) at the ASSS. Because gait rehabilitation is one of the main priorities in stroke rehabilitation, meta-analysis had a publication bias in favor of younger stroke rehabilitation patients included in the gait rehabilitation studies. Heterogeneity decreased slightly (*I*^2^ 98.7%; test *Q* significant *p* < 0.0001 to *I*^2^ 70%; test *Q* significant *p* = 0.0163) for the subgroup of rehabilitation patients. However, considering the heterogeneity of the subacute poststroke population, a selection bias in each study can be assumed. To limit the heterogeneity of WS assessments, we chose as primary criterion short-distance WS according the ten-meter walking test, which is considered the gold standard after stroke (22). For short-distance WS, we excluded other assessments ranging from the two-meter to the six-meter walking test and barefoot assessments. The ten-meter walking test is characterized by excellent reliability (2, 42, 60) and good sensitivity to change (61). Relationships between balance, cognition, autonomy and gait skills could not be assessed on secondary objectives because of missing data. In a recent meta-analysis of distance-limited walking tests after stroke, gait performance assessed by the ten-meter walking test was correlated with measures of strength, balance and physical activity (*r* = 0.26–0.8, *p* < 0.05) (22). While the relationship between gait skills and cognitive ability had been assessed in the general population over the age of 65 years (62) and in acute stroke patients (63), it needs to be completed at the subacute stage of stroke in order to advance in the analysis. The result of the meta-analysis of long-distance WS was very unstable, so it must be put into perspective.

The strength of this review is its originality because no recent review has been published on the gold standard of WS at the ASSS. This is of special interest because the functional prognosis of stroke has been revolutionized by recent therapies in stroke units, in the thrombolysis and thrombectomy era (27, 28, 64). However, WS must be one of the priority objectives of stroke rehabilitation to increase walking function (2), as well as survival (21), motor and balance functions, autonomy through community ambulation (61), social participation and health-related quality of life (54, 60).

Perspectives of this review are essential for patients because gait and balance disorders are the leading complaint after stroke (65). The ten-meter walking test is very relevant to human functioning, rehabilitation outcomes and patient-centered goals (22). Gait performance is poor in the ASSS, so it is essential to focus on WS improvement, which impacts all aspects of international classification of functioning disability and health, including quality of life and life expectancy (21, 22, 50, 61, 66). Therefore, to avoid the limit of missing data, further studies should collect systematic homogeneous data on scales assessing motor, balance, autonomy, quality of life and cognitive functions to analyze their relationship after subacute stroke (67), and also key outcomes data (e.g., early death, fatal intracranial hemorrhage and poor functional outcome). This meta-analysis studied WS during a simple gait task, while other studies examined WS links to balance and cognition with a focus on cognitive motor interference during gait: WS during dual task (68).

In conclusion, this first recent meta-analysis of WS highlighted a significant impairment of simple task gait performance at the ASSS. Gait skill is the main complaint of patients and one of the primary goals of stroke rehabilitation. Gait rehabilitation applies complementary means through analytical but also functional gait training techniques in order to recover efficient gait in all conditions of daily life. It is essential to further explore all aspects of gait skills to increase autonomy, social participation, quality of life and life expectancy after stroke.

## Author contributions

All authors listed have made a substantial, direct, and intellectual contribution to the work and approved it for publication.

## Conflict of interest

The authors declare that the research was conducted in the absence of any commercial or financial relationships that could be construed as a potential conflict of interest.

## Publisher's note

All claims expressed in this article are solely those of the authors and do not necessarily represent those of their affiliated organizations, or those of the publisher, the editors and the reviewers. Any product that may be evaluated in this article, or claim that may be made by its manufacturer, is not guaranteed or endorsed by the publisher.

## References

[1] Schnitzler A Woimant F Tuppin P de Peretti C. Prevalence of self-reported stroke and disability in the french adult population: a transversal study. PLoS ONE. (2014) 9:e115375. 10.1371/journal.pone.0115375 25521057 PMC4270760

[2] Perry J Garrett M Gronley JK Mulroy SJ. Classification of walking handicap in the stroke population. Stroke. (1995) 26:982–9. 10.1161/01.STR.26.6.982 7762050

[3] Tasseel-Ponche S Barbay M Roussel M Lamrani A Sader T Arnoux-Courselle A. Determinants of disability at 6 months after stroke: The GRECogVASC Study. Eur J Neurol. (2022) 29:1972–82. 10.1111/ene.15319 35276029

[4] Duarte E Marco E Muniesa JM Belmonte R Aguilar JJ Escalada F. Early detection of non-ambulatory survivors six months after stroke. NeuroRehabilitation. (2010) 26:317–23. 10.3233/NRE-2010-0568 20555154

[5] Jarvis HL Brown SJ Price M Butterworth C Groenevelt R Jackson K. Return to employment after stroke in young adults: how important is the speed and energy cost of walking? Stroke. (2019) 50:3198–204. 10.1161/STROKEAHA.119.025614 31554503 PMC6824505

[6] Jørgensen HS Nakayama H Raaschou HO Olsen TS. Recovery of walking function in stroke patients: the Copenhagen Stroke Study. Arch Phys Med Rehabil. (1995) 76:27–32. 10.1016/S0003-9993(95)80038-7 7811170

[7] Cho KH Lee JY Lee KJ Kang EK. Factors related to gait function in post-stroke patients. J Phys Ther Sci. (2014) 26:1941–4. 10.1589/jpts.26.1941 25540503 PMC4273063

[8] Hausdorff JM. Gait variability: methods, modeling and meaning. J Neuroengineering Rehabil. (2005) 2:19. 10.1186/1743-0003-2-19 16033650 PMC1185560

[9] Tasseel-Ponche S Yelnik AP Bonan IV. Motor strategies of postural control after hemispheric stroke. Neurophysiol Clin Neurophysiol. (2015) 45:327–33. 10.1016/j.neucli.2015.09.003 26520051

[10] Beyaert C Vasa R Frykberg GE. Gait post-stroke: pathophysiology and rehabilitation strategies. Neurophysiol Clin. (2015) 45:335–55. 10.1016/j.neucli.2015.09.005 26547547

[11] Roussel M Martinaud O Hénon H Vercelletto M Bindschadler C Joseph PA. The behavioral and cognitive executive disorders of stroke: the GREFEX Study. PLoS ONE. (2016) 11:e0147602. 10.1371/journal.pone.0147602 26824746 PMC4732595

[12] Yogev-Seligmann G Hausdorff JM Giladi N. The role of executive function and attention in gait. Mov Disord. (2008) 23:329–42. 10.1002/mds.21720 18058946 PMC2535903

[13] Barbay M Taillia H Nédélec-Ciceri C Bompaire F Bonnin C Varvat J. Prevalence of poststroke neurocognitive disorders using National Institute of Neurological Disorders and Stroke-Canadian Stroke Network, VASCOG criteria (vascular behavioral and cognitive disorders), and optimized criteria of cognitive deficit. Stroke. (2018) 49:1141–7. 10.1161/STROKEAHA.117.018889 29643258

[14] Weerdesteijn VG Niet MD Van Duijnhoven HJ Geurts AC. Falls in individuals with stroke. J Rehabil Res Dev. (2008) 45:1195–214. 10.1682/JRRD.2007.09.0145 19235120

[15] Tilson JK Wu SS Cen SY Feng Q Rose DR Behrman AL. Characterizing and identifying risk for falls in the LEAPS study: a randomized clinical trial of interventions to improve walking poststroke. Stroke. (2012) 43:446–52. 10.1161/STROKEAHA.111.636258 22246687 PMC3265675

[16] Chevreul K Durand-Zaleski I Gouépo A Fery-Lemonnier E Hommel M Woimant F. Cost of stroke in France. Eur J Neurol. (2013) 20:1094–100. 10.1111/ene.12143 23560508

[17] Kwakkel G Lannin NA Borschmann K English C Ali M Churilov L. Standardized measurement of sensorimotor recovery in stroke trials: consensus-based core recommendations from the stroke recovery and rehabilitation roundtable. Neurorehabil Neural Repair. (2017) 31:784–92. 10.1177/1545968317732662 28934918

[18] Fulk GD Echternach JL. Test-retest reliability and minimal detectable change of gait speed in individuals undergoing rehabilitation after stroke. J Neurol Phys Ther JNPT. (2008) 32:8–13. 10.1097/NPT0b013e31816593c0 18463550

[19] Mayo NE Wood-Dauphinee S Ahmed S Gordon C Higgins J McEwen S. Disablement following stroke. Disabil Rehabil. (1999) 21:258–68. 10.1080/096382899297684 10381238

[20] Fritz S Lusardi M. White paper: ≪ walking speed: the sixth vital sign ≫. J Geriatr Phys Ther 2001. (2009) 32:46–9. 10.1519/00139143-200932020-00002 20039582

[21] Chiu HT Wang YH Jeng JS Chen BB Pan SL. Effect of functional status on survival in patients with stroke: is independent ambulation a key determinant? Arch Phys Med Rehabil. (2012) 93:527–31. 10.1016/j.apmr.2011.10.018 22265084

[22] Cheng DKY Dagenais M Alsbury-Nealy K Legasto JM Scodras S Aravind G. Distance-limited walk tests post-stroke: a systematic review of measurement properties. NeuroRehabilitation. (2021) 48:413–39. 10.3233/NRE-210026 33967070 PMC8293643

[23] Salbach NM O'Brien K Brooks D Irvin E Martino R Takhar P. Speed and distance requirements for community ambulation: a systematic review. Arch Phys Med Rehabil. (2014) 95:117–28.e11. 10.1016/j.apmr.2013.06.017 23820298

[24] Schmid A Duncan PW Studenski S Lai SM Richards L Perera S. Improvements in speed-based gait classifications are meaningful. Stroke. (2007) 38:2096–100. 10.1161/STROKEAHA.106.475921 17510461

[25] Richards O. Hemiparetic gait following stroke. Part II: recovery and physical therapy. Gait Post. (1996) 4:149–62. 10.1016/0966-6362(96)01064-8

[26] Lang CE Bland MD Connor LT Fucetola R Whitson M Edmiaston J. The brain recovery core: building a system of organized stroke rehabilitation and outcomes assessment across the continuum of care. J Neurol Phys Ther JNPT. (2011) 35:194–201. 10.1097/NPT.0b013e318235dc07 22027474

[27] Wardlaw JM Zoppo G Yamaguchi T Berge E. Thrombolysis for acute ischaemic stroke. Cochrane Database Syst Rev. (2003) 3:CD000213. 10.1002/14651858.CD000213 12917889

[28] O'Rourke K Berge E Walsh C Kelly P. Percutaneous vascular interventions for acute ischaemic stroke. Cochrane Database Syst Rev. (2010) 6:CD007574. 10.1002/14651858.CD007574.pub2 20927761

[29] Moher D Liberati A Tetzlaff J Altman DG. Preferred reporting items for systematic reviews and meta-analyses: the PRISMA statement. PLoS Med. (2009) 6:6. 10.1371/journal.pmed.1000097 21603045 PMC3090117

[30] DerSimonian R Laird N. Meta-analysis in clinical trials. Control Clin Trials. (1986) 7:177–88. 10.1016/0197-2456(86)90046-2 3802833

[31] Cochran W. The combination of estimates from different experiments. Biometrics. (1954) 10:101–29. 10.2307/3001666

[32] Egger M Smith GD Schneider M Minder C. Bias in meta-analysis detected by a simple, graphical test. Bmj. (1997) 315:629–34. 10.1136/bmj.315.7109.629 9310563 PMC2127453

[33] Chua J Culpan J Menon E. Efficacy of an electromechanical gait trainer poststroke in Singapore: a randomized controlled trial. Arch Phys Med Rehabil. (2016) 97:683–90. 10.1016/j.apmr.2015.12.025 26802969

[34] Oostra K Oomen A Vanderstraeten G Vingerhoets G. Influence of motor imagery training on gait rehabilitation in sub-acute stroke: a randomized controlled trial. J Rehabil Med. (2015) 47:204–9. 10.2340/16501977-1908 25403275

[35] Conesa L Costa Ú Morales E Edwards DJ Cortes M León D. An observational report of intensive robotic and manual gait training in sub-acute stroke. J NeuroEngineering Rehabil. (2012) 9:13. 10.1186/1743-0003-9-13 22329866 PMC3305481

[36] Kollen B Kwakkel G Lindeman E. Hemiplegic gait after stroke: is measurement of maximum speed required? Arch Phys Med Rehabil. (2006) 87:358–63. 10.1016/j.apmr.2005.11.007 16500169

[37] Brincks J Nielsen JF. Increased power generation in impaired lower extremities correlated with changes in walking speeds in sub-acute stroke patients. Clin Biomech. (2012) 27:138–44. 10.1016/j.clinbiomech.2011.08.007 21899933

[38] Mak M Lau K. Speed-dependent treadmill training is effective to improve gait and balance performance in patients with sub-acute stroke. J Rehabil Med. (2011) 43:709–13. 10.2340/16501977-0838 21698340

[39] Persson C Hansson P Sunnerhagen K. Clinical tests performed in acute stroke identify the risk of falling during the first year: postural stroke study in Gothenburg (POSTGOT)^*^. J Rehabil Med. (2011) 43:348–53. 10.2340/16501977-0677 21267528

[40] Duncan P Studenski S Richards L Gollub S Lai SM Reker D. Randomized clinical trial of therapeutic exercise in subacute stroke. Stroke. (2003) 34:2173–80. 10.1161/01.STR.0000083699.95351.F2 12920254

[41] Wade DT Hewer RL. Functional abilities after stroke: measurement, natural history and prognosis. J Neurol Neurosurg Psychiatry. (1987) 50:177–82. 10.1136/jnnp.50.2.177 3572432 PMC1031489

[42] Rossier P Wade DT. Validity and reliability comparison of 4 mobility measures in patients presenting with neurologic impairment. Arch Phys Med Rehabil. (2001) 82:9–13. 10.1053/apmr.2001.9396 11239279

[43] Eng JJ Chu KS Dawson AS Kim CM Hepburn KE. Functional walk tests in individuals with stroke: relation to perceived exertion and myocardial exertion. Stroke. (2002) 33:756–61. 10.1161/hs0302.104195 11872900

[44] The LEAPS Investigative Team Duncan PW Sullivan KJ Behrman AL Azen SP Wu SS. Protocol for the locomotor experience applied post-stroke (LEAPS) trial: a randomized controlled trial. BMC Neurol. (2007) 7:39. 10.1186/1471-2377-7-39 17996052 PMC2222229

[45] Wade DT Wood VA Heller A Maggs J Langton Hewer R. Walking after stroke. Measurement and recovery over the first 3 months. Scand J Rehabil Med. (1987) 19:25–30. 3576138

[46] Mattlage AE Redlin SA Rippee MA Abraham MG Rymer MM Billinger SA. Use of accelerometers to examine sedentary time on an acute stroke unit. J Neurol Phys Ther JNPT. (2015) 39:166–71. 10.1097/NPT.0000000000000092 26035120 PMC4470858

[47] ATS Committee on Proficiency Standards for Clinical Pulmonary Function Laboratories. ATS statement: guidelines for the six-minute walk test. Am J Respir Crit Care Med. (2002) 166:111–7. 10.1164/ajrccm.166.1.at1102 12091180

[48] Pohl PS. Influence of stroke-related impairments on performance in 6-minute walk test. J Rehabil Res Dev. (2002) 39:439–44. 17638141

[49] Troosters T Gosselink R Decramer M. Six-minute walk test: a valuable test, when properly standardized. Phys Ther. (2002) 82:826–7. 10.1093/ptj/82.8.826 12147012

[50] Fulk GD Ludwig M Dunning K Golden S Boyne P West T. Estimating clinically important change in gait speed in people with stroke undergoing outpatient rehabilitation. J Neurol Phys Ther. (2011) 35:82–9. 10.1097/NPT.0b013e318218e2f2 21934363

[51] Xiao X Lin Q Lo WL Mao YR Shi X Cates RS. Cerebral reorganization in subacute stroke survivors after virtual reality-based training: a preliminary study. Behav Neurol. (2017) 2017:6261479. 10.1155/2017/6261479 28720981 PMC5506482

[52] Preston E Ada L Stanton R Mahendran N Dean CM. Prediction of independent walking in people who are nonambulatory early after stroke: a systematic review. Stroke. (2021) 52:3217–24. 10.1161/STROKEAHA.120.032345 34238016

[53] Bohannon RW Williams Andrews A. Normal walking speed: a descriptive meta-analysis. Physiotherapy. (2011) 97:182–9. 10.1016/j.physio.2010.12.004 21820535

[54] Khanittanuphong P Tipchatyotin S. Correlation of the gait speed with the quality of life and the quality of life classified according to speed-based community ambulation in Thai stroke survivors. NeuroRehabilitation. (2017) 41:135–41. 10.3233/NRE-171465 28527227

[55] Fulk GD Echternach JL Nof L O'Sullivan S. Clinometric properties of the six-minute walk test in individuals undergoing rehabilitation poststroke. Physiother Theory Pract. (2008) 24:195–204. 10.1080/09593980701588284 18569856

[56] Tang A Sibley KM Bayley MT McIlroy WE Brooks D. Do functional walk tests reflect cardiorespiratory fitness in sub-acute stroke? J NeuroEngineering Rehabil. (2006) 3:23. 10.1186/1743-0003-3-23 17010203 PMC1592502

[57] Lecoffre C de Peretti C Gabet A Grimaud O Woimant F Giroud M. National trends in patients hospitalized for stroke and stroke mortality in France, 2008 to 2014. Stroke. (2017) 48:2939–45. 10.1161/STROKEAHA.117.017640 28970279

[58] Béjot Y Daubail B Giroud M. Epidemiology of stroke and transient ischemic attacks: current knowledge and perspectives. Rev Neurol. (2016) 172:59–68. 10.1016/j.neurol.2015.07.013 26718592

[59] GBD 2019 Stroke Collaborators. Global, regional, and national burden of stroke and its risk factors, 1990-2019: a systematic analysis for the Global Burden of Disease Study 2019. Lancet Neurol. (2021) 20:795–820. 10.1016/S1474-4422(21)00252-0 34487721 PMC8443449

[60] Collen FM Wade DT Bradshaw CM. Mobility after stroke: reliability of measures of impairment and disability. Int Disabil Stud. (1990) 12:6–9. 10.3109/03790799009166594 2211468

[61] Salbach NM Mayo NE Higgins J Ahmed S Finch LE Richards CL. Responsiveness and predictability of gait speed and other disability measures in acute stroke. Arch Phys Med Rehabil. (2001) 82:1204–12. 10.1053/apmr.2001.24907 11552192

[62] Garcia-Pinillos F Cozar-Barba M Munoz-Jimenez M Soto-Hermoso V Latorre-Roman P. Gait speed in older people: an easy test for detecting cognitive impairment, functional independence, and health state: Gait speed and older people. Psychogeriatrics. (2016) 16:165–71. 10.1111/psyg.12133 26114989

[63] Sagnier S Renou P Olindo S Debruxelles S Poli M Rouanet F. Gait change is associated with cognitive outcome after an acute ischemic stroke. Front Aging Neurosci. (2017) 9:153. 10.3389/fnagi.2017.00153 28572768 PMC5435741

[64] Katan M Luft A. Global burden of stroke. Semin Neurol. (2018) 38:208–11. 10.1055/s-0038-1649503 29791947

[65] de Peretti C Grimaud O Tuppin P Chin F Woimant F. Prévalence des accidents vasculaires cérébraux et de leurs séquelles et impact sur les activités de la vie quotidienne: apports des enquêtes déclaratives Handicap-santé-ménages et Handicap-santé-institution. Bull Epidemiol Hebd. (2012) 10:1–6.

[66] Winovich DT Longstreth WT Arnold AM Varadhan R Zeki Al Hazzouri A Cushman M. Factors associated with ischemic stroke survival and recovery in older adults. Stroke. (2017) 48:1818–26. 10.1161/STROKEAHA.117.016726 28526765 PMC5553701

[67] Salinas J Sprinkhuizen SM Ackerson T Bernhardt J Davie C George MG. An international standard set of patient-centered outcome measures after stroke. Stroke. (2016) 47:180–6. 10.1161/STROKEAHA.115.010898 26604251 PMC4689178

[68] Park MO Lee SH. Effect of a dual-task program with different cognitive tasks applied to stroke patients: a pilot randomized controlled trial. NeuroRehabilitation. (2019) 44:239–49. 10.3233/NRE-182563 31006694

